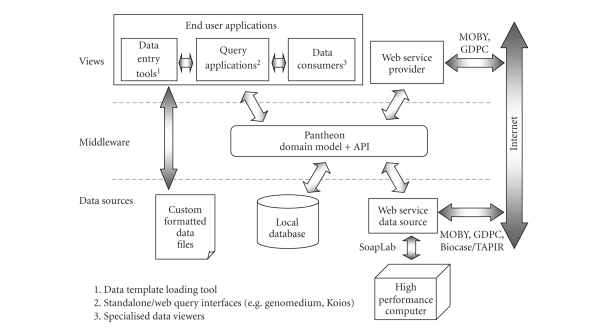# The Generation Challenge Programme Platform: Semantic Standards and Workbench for Crop Science

**DOI:** 10.1155/2008/369601

**Published:** 2008-04-30

**Authors:** Richard Bruskiewich, Martin Senger, Guy Davenport, Manuel Ruiz, Mathieu Rouard, Tom Hazekamp, Masaru Takeya, Koji Doi, Kouji Satoh, Marcos Costa, Reinhard Simon, Jayashree Balaji, Akinnola Akintunde, Ramil Mauleon, Samart Wanchana, Trushar Shah, Mylah Anacleto, Arllet Portugal, Victor Jun Ulat, Supat Thongjuea, Kyle Braak, Sebastian Ritter, Alexis Dereeper, Milko Skofic, Edwin Rojas, Natalia Martins, Georgios Pappas, Ryan Alamban, Roque Almodiel, Lord Hendrix Barboza, Jeffrey Detras, Kevin Manansala, Michael Jonathan Mendoza, Jeffrey Morales, Barry Peralta, Rowena Valerio, Yi Zhang, Sergio Gregorio, Joseph Hermocilla, Michael Echavez, Jan Michael Yap, Andrew Farmer, Gary Schiltz, Jennifer Lee, Terry Casstevens, Pankaj Jaiswal, Ayton Meintjes, Mark Wilkinson, Benjamin Good, James Wagner, Jane Morris, David Marshall, Anthony Collins, Shoshi Kikuchi, Thomas Metz, Graham McLaren, Theo van Hintum

**Affiliations:** ^1^Crop Research Informatics Laboratory, International Rice Research Institute (IRRI), DAPO Box 7777, Metro Manila, Philippines; ^2^Crop Research Informatics Laboratory, International Maize and Wheat Improvement Center (CIMMYT), Apdo. Postal 6-641, 06600 Mexico, DF, Mexico; ^3^Centre International de Recherche Agronomique pour le Développement (CIRAD), Avenue Agropolis, 34398 Montpellier, Cedex 5, France; ^4^Bioversity International, Via dei Tre Denari 472/a, 00057 Maccarese (Fiumicino), Rome, Italy; ^5^National Institute for Agrobiological Sciences (NIAS), Kannondai 2-1-2, Tsukuba, Ibaraki 305-8602, Japan; ^6^Empresa Brasileira de Pesquisa Agropecuaria (EMBRAPA), Parque Estação Biologia Final W5 Norte, 70770-900 Brasilia, DF, Brazil; ^7^Centro Internacional de la Papa (CIP), Avenida La Molina 1895, La Molina, Apartado Postal 1558, Lima 12, Peru; ^8^International Crops Research Institute for the Semi-Arid Tropics, Patancheru, Andhra Pradesh 502324, India; ^9^International Center for Agricultural Research in the Dry Areas, P.O. Box 5466, Aleppo, Syria; ^10^National Center for Genetic Engineering and Biotechnology, 113 Thailand Science Park, Phahonyothin Road, Klong 1, Klong Luang, Pathumthani 12120, Thailand; ^11^Institute of Computer Science, College of Arts and Sciences, University of the Philippines, Los Baños, Laguna 4031, Philippines; ^12^Department of Computer Science, University of the Philippines, Room 215, Melchor Hall, Diliman, Quezon City 1101, Philippines; ^13^National Center for Genome Resources, 2935 Rodeo Park Drive East, Santa Fe, NM 87505, USA; ^14^Scottish Crop Research Institute, Invergowrie, Dundee DD2 5DA, Scotland, UK; ^15^Department of Plant Breeding, Cornell University, Ithaca, NY 14853, USA; ^16^African Centre for Gene Technologies, P.O. Box 75011, Lynnwood Ridge 0040, South Africa; ^17^Department of Medical Genetics, Faculty of Medicine, The University of British Columbia, Vancouver, BC, Canada V6T 1Z3; ^18^School of Computing Science, Simon Fraser Universtiy, 8888 University Drive, Burnaby, BC, Canada V5A 1S6; ^19^Bioinformatics Graduate Program, Genome Sciences Centre, BC Cancer Agency, 100-570 West 7th Avenue, Vancouver, BC, Canada V5Z 4S6; ^20^Centre for Genetic Resources, The Netherlands (CGN), P.O. Box 16, 6700 AA Wageningen, The Netherlands

## Abstract

The Generation Challenge programme (GCP) is a global crop research consortium directed toward crop improvement through the application of comparative biology and genetic resources characterization to plant breeding. A key consortium research activity is the development of a GCP crop bioinformatics platform to support GCP research. This platform includes the following: (i) shared, public platform-independent domain models, ontology, and data formats to enable interoperability of data and analysis flows within the platform; (ii) web service and registry technologies to identify, share, and integrate information across diverse, globally dispersed data sources, as well as to access high-performance computational (HPC) facilities for computationally intensive, high-throughput analyses of project data; (iii) platform-specific middleware reference implementations of the domain model integrating a suite of public (largely open-access/-source) databases and software tools into a workbench to facilitate biodiversity analysis, comparative analysis of crop genomic data, and plant breeding decision making.

## 1. INTRODUCTION

The fast-moving fields of comparative genomics, molecular breeding, and bioinformatics have the potential to bring new knowledge to bear on problems encountered by resource-poor farmers. These problems include abiotic stresses (such as drought and soil salinity) and biotic stresses (such as plant diseases and pests). The Generation Challenge Programme (GCP; http://www.generationcp.org/) aims to exploit advances in molecular biology to harness the rich global heritage of plant genetic resources and contribute to a new generation of stress-tolerant varieties that meet the needs of these farmers and the consumers of their crops. The GCP brings together three sets of partners: member agricultural research institutes of the Consultative Group on International Agricultural Research (CGIAR; http://www.cgiar.org/), advanced research institutes in developed countries, and national agricultural research and extension systems in developing countries, to undertake a long-term program of globally integrated scientific research, capacity building, and delivery of products for the above goal.

Central to GCP activities is the development of an integrated platform of molecular biology and bioinformatics tools to be applied to the research objectives of the GCP. The resulting platform is also intended to be a “global public good” to be made freely available to all crop researchers and breeders around the world, thus enabling agricultural scientists, particularly in developing countries, to more readily apply information about elite genetic stocks, genomic knowledge, and new breeding technologies that are becoming available to their local breeding programmes.

The goal of this GCP crop informatics platform is to provide solutions for priority end-user needs for biodiversity analysis, comparative analysis of crop genomic data, and plant breeding decision making. Development of the platform is driven by the following observations:

- GCP partners (and the international crop research community in general) are globally distributed, each research team having relatively large datasets to share and integrated datasets that reside in diverse online, but locally curated databases;
- GCP research covers a diversity of crop species;
- GCP research spans a wide range of scientific data types, including germplasm, genomic, phenotypic, as well as crop physiological and geographic information, this constellation of data types is evolving with time as new experimental technologies are created;
- GCP scientists (and crop scientists in general) need to apply a wide range of analytical tools already used by their research communities; they also need new tools to meet new or evolving needs; integration of such tools to interoperate with one another is a nontrivial task.

A GCP crop information platform is being developed to better meet these challenges by managing genetic resources, genomics, and crop information using the following components:

- shared public platform-independent set of scientific domain models, ontology, and data templates to cross-link all data types and analysis processes within the platform;
- GCP domain model-constrained web service and registry technologies to identify, share, and manage the analysis of information, as well as to integrate it across a network of diverse globally dispersed data sources connected to the Internet;
- reference implementations of platform-specific middleware using the GCP domain model;
- a suite of open-source software tools (adopted or newly developed) integrated into a workbench and accessing web-connected data sources. Included in this suite is software to provide enhanced access to high-performance computational (HPC) grid facilities enabling computationally intensive and/or high-throughput analyses of project data.

This paper will survey progress on some of the central components of the platform, with a special emphasis on the domain model, a reference Java middleware implementation, and Internet protocol aspects of the project.

## 2. MATERIALS AND METHODS

### 2.1. GCP domain model

To cope with the scope, diversity, and dispersion of crop information, GCP researchers formulated a vision to specify a consensus blueprint of a scientific domain model and associated ontology. The resulting models and ontology allow a “model-driven architecture” for the development of GCP software and network protocols [1].

The domain model is documented in Unified Modeling Language (UML). Computable versions of the UML model are archived in the DemeterUML folder of the “Pantheon” project in CropForge (http://cropforge.org/) software project repository. The UML diagrams themselves are indexed and published with supporting narratives on a project website (http://pantheon.generationcp.org/demeter). The bulk of the models are specified with the UML <<interface>> stereotype.

At the heart of the domain model are generic core model interfaces from which other specific scientific model interfaces are derived. This core model starts with the concept of simple identification of data objects in the system (using the * SimpleIdentifier* interface), which is extended by several more specific interfaces. The core includes a general concept of *Entity*, which serves as the superclass for most other interfaces describing major scientific concepts or data types in the system. The *Entity* interface documents generic metadata about objects in the system, including specific annotation of object characteristics using a rich *Feature* model. Other packages in the core models provide utility models for ontology, publication, and experimental study management.

Additional scientific models are derived as extensions of the core models. For example, the base interface classes of most specific major concepts or experimental objects in the scientific domain of discourse of the GCP, such as *Germplasm*, * Map*, or *GeneProduct*, directly extend the *Entity* model, adding subdomain-specific attributes as required. More lightweight concepts in the system extend simpler interfaces such as *Feature*.

For the elaboration of specific components of the core, as well as scientific domain models, the project generally adapts extant public domain models. For example, the *Germplasm* and *Study* subdomain models are derived from the data models of the open-source International Crop Information System (ICIS, http://www.icis.cgiar.org/; [2–4]). Aspects of the genotype (and associated genetic map and genomic sequence) models are influenced by public initiatives such as the Chado relational database schemata of the Generic Model Organism Database (GMOD) project [5]. The production-release GCP domain model is being validated based on feedback from project scientists and developers, who are striving to validate the model by practical application in data management and platform implementation.

A significant feature of the domain model is the reliance on extensible controlled vocabulary and ontology (CVO) to define the full semantics of specialized types, feature attributes, and annotation values of instances of the model classes. Where possible, the GCP is simply adopting existing CVO standards, such as from the gene ontology [6], plant ontology [7], and Microarray Gene Expression Data Society (MGED) ontology [8] consortia. Where no appropriate ontology has yet been formalized, new dictionaries of terms are being compiled in collaboration with GCP scientists. CVO dictionaries selected for the platform are being catalogued in a dedicated online database (at http://pantheon.generationcp.org/) with web browser and web service access. Each selected dictionary is assigned a GCP ontology index number to facilitate platform management of the ontology. Where an existing public ontology already has its own accession identifiers (e.g., GO identifiers for the GO CVO), these identifiers are propagated into the full GCP identifier for the corresponding CVO terms. However, newly specified CVO lacking such a number space are assigned *de novo* GCP accession identifiers.

### 2.2. GCP platform middleware

Since a March 2006 public review of the GCP domain model, the GCP informatics team has developed selected technology-specific GCP implementations of the model, primarily focusing on Java-based middleware specifying a Model-View-Controller (MVC) architecture (see Figure 1). Although the primary development stream of the project is focusing on a Java language implementation, the GCP domain model is a “platform-independent model” amenable to implementation with other computing languages and is, indeed, being used to guide some complementary work with languages such as Perl, Javascript, and PHP. The Java-based middleware was given the overall name “Pantheon” to account for the usage of various ancient agricultural gods (mostly agricultural, e.g., Demeter, Ceres, Belenus, Osiris) in the naming of the various layers and component parts of the code base. This code base is open source and managed under the Pantheon project in CropForge.

In addition to a Java implementation of the GCP domain model, a Java application programming interface (API) was specified to assist with and standardize software integration of components within the middleware architecture. These interfaces are collected into a core Java library called “PantheonBase” hosted as a module in the Ceres section of Pantheon (under Ceres/projects/Pantheonbase). PantheonBase includes a simple *DataSource* interface for read-only query retrieval of data from any source (local or distributed); a *DataConsumer* interface to guide integration and synchronization of applications and viewers wishing to use data extracted using the middleware; and finally, a *DataTransformer* interface to provide a framework for analysis and transformations (e.g., reformatting) of data. PantheonBase was deliberately designed to be essentially agnostic about the GCP domain model per se, for maximum flexibility and possible reuse with non-GCP-compliant data.

Additional support libraries are being provided within Ceres to support GCP domain model-driven *DataSource* development. In addition to core and support libraries, the Pantheon project provides a clearinghouse for platform and data-type-specific components. These components include adapters implementing the *DataSource* interface for specific data sources (archived in Osiris) for various crop databases at various GCP partner and external sites. Among others, current *DataSource* implementations include a wrapper for the ICIS and for GMOD schemata (Chado, Gbrowse). Other Pantheon components provide application support, including a search engine, data visualization, and web service provider implementations (in Belenus). Examples of the latter are support for NCGR ISYS [9], support for stand-alone applications based on Eclipse/RCP [10], and a web-based GCP domain-model-compliant web-based search engine (Koios).

### 2.3. GCP network protocols

The GCP domain model is also being applied to platform-specific implementation of a GCP network based on Internet bioinformatics data exchange protocols such as BioMOBY [11], SoapLab [12], SSWAP [13], and Tapir [14]. In this paper, in the interest of brevity, we will discuss only BioMOBY, arepresentative protocol being used in the GCP network.

For BioMOBY, data types were designed using GCP domain model semantics. Although generally faithful in translating the semantics of the Demeter UML specification of the domain model (i.e., the *SimpleIdentifier* interface is represented as a *GCP_SimpleIdentifier* data type), the GCP BioMOBY data types simplify the data representation as a concession to BioMOBY design constraints and to web service performance.

One key example of this is the extensive substitution of *GCP_SimpleIdentifier* objects, instead of fully detailed data objects, at the end of model-to-model association edges found in the Demeter model. The rationale for this is the expectation that, in most cases, web services can apply a concept of “lazy loading” of data-type components, in which one identifies what objects might be embedded in a parent object, but does not necessarily retrieve their details until the user needs them (as a separate web service accepting a *GCP_SimpleIdentifier* of the object but returning the fully populated complex object of the specified type).

UML diagrams with supporting explanatory narration for these GCP-specific BioMOBY data types are published on the Pantheon website (http://pantheon.generationcp.org/moby), which is complemented by a website documenting GCP BioMOBY implementation details (http://moby.generationcp.org/). Supporting the BioMOBY protocol in Pantheon are a series of Pantheon modules for interconversion between GCP MOBY data types and Demeter-compliant Java objects, for web service provider implementation, and for a MOBY client *DataSource* adapter to communicate with GCP-compliant web service providers.

Using GCP model-constrained BioMOBY data types (all prefixed with “GCP_” in their name in the MOBY central registry), various GCP teams are deploying GCP-compliant web services from a common proposed list of documented web service use cases. Concurrently, the MOBY client *DataSource* adapter is being elaborated to communicate with these web services and import remote data into local “workbench” instances of the GCP platform.

### 2.4. Additional tools integrated into the GCP platform

The GCP domain model and associated platform middleware is not an end in itself. Rather, the goal of these informatics products is to serve as a semantically and operationally rich scaffold for the integration of both local and remote (Internet-connected) bioinformatics data resources and analysis tools.

In addition to data sources and tools already mentioned above, additional open-source third-party analysis tools already coded using Java, but agnostic concerning the GCP framework are being connected to the platform through targeted software engineering. To this end, GCP developers are connecting several public open-source applications by writing suitable *DataSource* adapters, *DataConsumer*, or *DataTransformer* integration code. These include Java software hosted by GMOD such as the Apollo genome browser [15], tools forming part of the Genomic Diversity and Phenotype Connection (GDPC) protocol such as Tassel [16], and tools such as TIGR Multiple Experiment Viewer [17] for microarray analysis, the Comparative Map and Trait Viewer [18] connected to the NCGR ISYS framework [9], the Cytoscape network visualization tool [20], and the MAXD microarray system [19].

## 3. RESULTS AND DISCUSSION

The GCP consortium was formally established in 2003. The first meeting of the bioinformatics and crop informatics development team of the GCP, designated as Subprogramme 4, was hosted in Rome, in February 2004. The general user needs and project goals were coarsely mapped out at this meeting, with some considerable differences in opinion voiced at how to construct the required informatics framework for the GCP. In May 2004, a smaller team of software experts met in Mexico to discuss project management, identify key user needs and platform requirements, and make some initial progress in the design of the system. Key decisions at this latter meeting were the adoption of the “model-driven architecture” paradigm for system development and to embrace web services as a key technology for global integration of systems. Numerous development meetings have been convened annually since these initial meetings to further refine and advance the design and implementation of the platform.

In particular, a milestone review of the GCP domain model and initial software systems using the model was held in Pretoria, South Africa in March 2006. Since that time, a number of early release versions of software systems based on GCP platform technology have become available, generally documented at http://pantheon.generationcp.org/ and publicly downloadable from various CropForge projects. A special “communications” project for GCP-specific projects is also available on CropForge at the http://cropforge.org/projects/gcpcomm to further inform prospective users on the variety of such GCP software tools now available, and provide a venue for user discussions and feedback about the tools.

### 3.1. So, what can I do with the GCP platform?

The vision of the platform development team of the bioinformatics and crop informatics subprogramme of the GCP is to establish a truly easy to use but extensible workbench providing interoperability and enhanced data access across all GCP partner sites and, later, across the global crop research community. As indicated above, the GCP domain model has a scope of data type coverage that spans most of the pertinent scientific data types found in crop research from upstream laboratory experiments through germplasm manipulations, in a georeferenced characterized field setting. The diversity of potential data sources and analysis tools is similarly large. What the platform facilitates is transparent data flows between such data sources and tools, whether from locally administered databases or remote Internet-connection resources.

In this light, a number of practical “use cases” may be described in general terms, as a series of data manipulation steps, to highlight some of the anticipated usage of the platform. As an indication of the data retrieval and analysis scope of the GCP platform, we describe a general integrative use case here below, in terms of a series of defined steps.

**General GCP platform analysis use case for crop improvement**

1. Retrieve the list of all genetic maps that include a quantitative trait locus (QTL) for a specified trait.
2. Retrieve selected maps in the list, from a project database or source file containing such maps.
3. Load this into a suitable mapping tool (e.g., the comparative map and trait visualization tool, CMTV).
4. Extract the pairs of flanking markers for the QTL.
5. From a second (crop) database, retrieve the list of all germplasm that have been genotyped with these flanking markers.
6. Retrieve all the pertinent passport, genotype, and phenotype information about the germplasm in the list.
7. In parallel to the steps (5) and (6), if available, retrieve any gene locus candidates within (genetic/physical/sequence) map intervals which are defined by flanking markers which are molecular sequence based.
8. Retrieve gene functional information about the gene loci compiled in step (7).
9. Retrieve the alleles of “interesting” genes from (8), in the list of germplasm identified in step (5).
10. Plot germplasm passport, genotype, and phenotype information on geographical information maps.
11. Retrieve information about the environmental characteristics of the geographical regions identified in step (10).
12. Identify germplasm, for further detailed evaluation, which appears to be adapted to target environments, which have promising phenotypic values identified in step (6) and which contains target alleles of gene loci identified in step (9).
13. Identify genotyping (marker) systems potentially available from step (9), for marker assisted selected transfer of target traits from identified germplasm to additional germplasm targets.

## 4. CONCLUSIONS

The vision of the platform development team of the bioinformatics and crop informatics subprogramme of the GCP is to establish a state-of-art but truly easy-to-use and extensible open-source workbench providing interoperability and enhanced data access across all GCP partner sites and, by extension, the global crop research community.

Although several attempts have been made in the past to build such globally integrative bioinformatics systems, few have the global distribution of partners, scope of crop research, diversity of data types, and magnitude of datasets in comparison to the GCP consortium, nor do they have the long-term project perspective of 10 years. In addition, the GCP platform is specifically targeted to bioinformatics for developing world crop research, in contrast to biomedical research, and also strives to integrate databases from many plants and crops less well represented by well-funded model organisms and crops.

In these respects, the GCP platform effort represents an extremely ambitious but very useful global public good resource for crop research. It is still conceded to be, in several respects, an incomplete evolving product, one with many rough edges and incompletely met end-user needs; however, the open-source and public nature of the project provides a credible venue for wide participation of interested developers and prospective end users in the future evolution and deployment of the platform.

## Figures and Tables

**Figure 1:**